# An Efficient Wireless Recharging Mechanism for Achieving Perpetual Lifetime of Wireless Sensor Networks

**DOI:** 10.3390/s17010013

**Published:** 2016-12-23

**Authors:** Hongli Yu, Guilin Chen, Shenghui Zhao, Chih-Yung Chang, Yu-Ting Chin

**Affiliations:** 1 School of Computer and Information Engineering, Chuzhou University, Chuzhou 239000, China; hly@chzu.edu.cn (H.Y.); glchen@chzu.edu.cn (G.C.); shzhao@ah.edu.cn (S.Z.); 2 Department of Computer Science and Information Engineering, Tamkang University, New Taipei City 25137, Taiwan; 143221@mail.tku.edu.tw

**Keywords:** wireless sensor network, energy management, lifetime, energy recharging efficiency, recharging path reduction

## Abstract

Energy recharging has received much attention in recent years. Several recharging mechanisms were proposed for achieving perpetual lifetime of a given Wireless Sensor Network (WSN). However, most of them require a mobile recharger to visit each sensor and then perform the recharging task, which increases the length of the recharging path. Another common weakness of these works is the requirement for the mobile recharger to stop at the location of each sensor. As a result, it is impossible for recharger to move with a constant speed, leading to inefficient movement. To improve the recharging efficiency, this paper takes “recharging while moving” into consideration when constructing the recharging path. We propose a Recharging Path Construction (RPC) mechanism, which enables the mobile recharger to recharge all sensors using a constant speed, aiming to minimize the length of recharging path and improve the recharging efficiency while achieving the requirement of perpetual network lifetime of a given WSN. Performance studies reveal that the proposed RPC outperforms existing proposals in terms of path length and energy utilization index, as well as visiting cycle.

## 1. Introduction

Wireless sensor networks (WSNs) have been widely used in various fields such as environmental monitoring, health care, industry, transport and logistics [1,2,3,4,5]. Most wireless sensors are battery-powered. The limited energy of the batteries is a constraint on the lifetime of WSNs. Thus, the issue of energy management has received much attention in the last decade. In the literature, plenty of approaches have been proposed to cope with the energy management problem. These studies mainly focus on two techniques: energy conservation technology [6,7,8,9], and energy replenishment technology [10,11,12,13,14,15,16].

The energy conservation technology aims to prolong the lifetime of WSNs by reducing the energy consumption of the network. In the past years, some energy conservation algorithms [6,7,8,9] were proposed. To extend the lifetime of WSNs, most proposed mechanisms use power reduction to conserve the limited battery energy. These mechanisms include the optimization of routing decisions, node energy management, MAC protocols, cross-layer optimization, etc. However, since the energy conservation approaches only try to reduce energy consumption, without considering the energy replenishment, it is difficult to sustain the operations of WSNs.

Energy replenishment technology involves recharging the sensor by collecting energy from the surroundings or RF-based energy transmission, aiming to achieve perpetual network operation. There are numerous proposed schemes [10,11,12,13,14,15,16,17,18,19] to recharge the sensors in the monitoring area. Depending on the source of recharging energy, the existing energy replenishment mechanisms can be further classified into two categories: energy replenishment from environmental energy or from mobile rechargers.

In the first class, numerous energy harvesting systems [10,11,12,13] have been proposed. They consider that there are various renewable environment resources, such as solar energy, wind energy, thermal energy, etc. Since these renewable energy sources are mainly obtained from the environment, these energy supports are unreliable.

To improve the instability characteristics usually found in the first class, plenty of RF-based energy transmission mechanisms have been proposed [14,15,16]. They assume that the sensors are stationary in the network. The sink node is considered as a static energy station which provides energy to a mobile recharger. Then, these mechanisms employ several rechargers to periodically visit and provide energy to each sensor. This implies that the sensors can be recharged at fixed time intervals, however, how to improve the energy efficiency while maintaining the recharging demand of each sensor is still a big challenge.

This paper considers the problem of energy recharging efficiency of wireless sensor networks. We present an algorithm to construct a path which passes through each sensor for a mobile recharger to recharge each sensor with a guarantee that each sensor can be fully recharged. To reduce the path length, the proposed algorithm then utilizes the triangle theorem, aiming at minimizing the recharging path length. The contributions of this paper are itemized as follows:

(1) *Recharging while moving*:
  This paper presents and implements the concept of “recharging while moving”. The mobile recharger therefore can efficiently move along the path with a constant speed.
(2) *Guarantee that each sensor can be fully recharged*:
  A recharging segment is analyzed and constructed such that the mobile recharger moving along the segment of each sensor can guarantee that each sensor is fully recharged.
(3) *Joint mobility and energy recharging*:
  As far as we know, this is the first work that allows the recharger to be moved with a constant speed while each sensor can be fully recharged by mobile recharger.
(4) *Reducing the length of recharging path*:
  The proposed path reduction approach further reduces the length of recharging path while satisfying the perpetual operation demand of WSNs, as compared with existing works [14,15,16].

The remainder of this paper is organized as follows: Section 2 reviews related works on energy replenishment. Section 3 presents the network environment and problems investigated in this paper. Section 4 gives a sensor recharging model which is applied in the proposed RPC mechanism. In Section 5, the performance evaluation of the proposed RPC algorithm is presented. Section 6 concludes this paper.

## 2. Related Works

This section reviews existing works related to energy replenishment in WSNs. In the literature, plenty of mechanisms have been proposed to support perpetual network operations. These solutions can be classified into two categories, including the energy replenishment by environmental energy resources and energy replenishment by mobile rechargers. The following reviews these related studies.

### 2.1. Energy Replenishment by the Environmental Energy Resources

There are numerous studies focus on how to transform renewable energy, such as solar energy, wind power and thermal energy, into electrical energy for maintaining the perpetual operations of WSNs.

In [10], Jay et al. proposed a micro-solar power subsystem to supply energy to sensors. This system consists of several pieces, including solar panels, regulators and energy storage elements. The solar panel acquires the solar energy from the environment first. Then, the system transforms this solar energy into electric energy in order to recharge the sensors. However, to ensure the sensors can be recharged constantly, the micro-solar power subsystem must be connected to each sensor, which increases the size of each sensor. Furthermore, the fatal shortcoming of solar energy is its unreliability factor, as the strength of light changes with the weather, so the energy provided to sensors is unstable.

As a kind of available renewable and free energy source, wind energy has been widely used in supplementary energy systems. Tan et al. [11] provided a wind turbine generator (WTG) to sense the wind speed of the environment. This wind energy harvesting (WEH) system transforms the wind energy into electrical energy to recharge the sensors. However, as the strength of wind is unstable, the WEH could not obtain the expected energy. In addition, the size of the WEH mechanics may pose a new deployment problem.

The concept of using a thermal energy harvesting system to recharge WSNs has received significant attention over the past years. Study [12] proposed a Seebeck heat pump to transform the surrounding thermal energy into electric energy. The proposed device was composed of two thermoelectric generator (TEG) systems: an energy collection system and an energy recharging system. The energy collection system captures the solar radiation while the energy recharging system recharges the sensor batteries. However, the construction of the proposed TEGs system is too complex. Additionally, the energy consumption of the TEG system is higher than that of other energy harvesting systems [11].

### 2.2. Energy Replenishment by Mobile Rechargers

Since the amount of energy that can be harvested from the environment is limited, numerous studies [13,14,15,16] have focused on how to recharge sensors by using a mobile recharger. He et al. [13] proposed an energy recharging scheme based on RFID technology. In this study, the tag is considered as a sensor which obtains energy from the reader through RF signals. However, the main problem is how to deploy the readers to guarantee the tags will be fully recharged while minimizing the energy cost. In addition, since each sensor needs a RF reader, the number of readers increases with the number of sensors, resulting in a high cost of the recharging system.

To resolve the problems of [13], Zhang et al. designed a novel recharging paradigm, called collaborative mobile charging, which recharges the sensors in the monitoring area by using several mobile rechargers [14]. The mechanism assumes the rechargers are able to charge each other. Then, a multiple mobile recharger collaboration schedule is proposed to recharge the sensors in the WSNs. However, the mobile recharger can only recharge one sensor in a certain time period, leading to low recharging efficiency.

With the purpose of reducing the number of mobile rechargers needed, reference [15] proposed an energy recharging system based on a single mobile recharger. This system consists of three parts, including a mobile recharger, sensors with power receivers, and an energy station. The energy station arranges the visit sequence for the mobile recharger according to the energy consumption information reported by the sensors. Then, the mobile recharger recharges the sensors following the arranged sequence. However, the sensors need to transmit their energy information to the energy station periodically, leading to additional energy consumption. Furthermore, they do not consider the recharging path length of mobile recharger, resulting in low recharging efficiency.

To improve the recharging efficiency of mobile recharging systems, Xie et al. [16] considered the requirement of periodic recharging of the sensors and proposed a mobile recharging algorithm by using a wireless charging vehicle (WCV). They assume that the WCV carries a power station. The WCV travels over the WSNs and recharges the sensors periodically. The travel path of the WCV is constructed by applying the shortest Hamiltonian cycle. Although the recharging path length has been considered, the path length can be further reduced.

Shi et al. proposed a recharging path construction mechanism based on the shortest Hamiltonian cycle [18]. The constructed path passes through the location of each sensor. A wireless charging vehicle travels along the path to recharge sensors. However, the path length still can be further reduced.

In [19], a recharging mechanism, called OWER-MDG is proposed. A mobile vehicle (SenCar) is used to recharge sensors and collect data from them. In each run, OWER-MDG selects several anchor sensors from among the static sensors and constructs a path passing through the recharging ranges of the anchor sensors. The sensors with low remaining energy will be selected as anchor sensors and will be recharged before other sensors. Since the path constructed in each run cannot visit all sensors, several runs are needed to fully recharge all sensors in the monitoring area. That is, the path that visits all sensors can be treated as the connection of the paths constructed in several runs. Though the data collection can be completed in each run, the path for mobile recharger to recharge all sensors is long.

All of the energy management mechanisms discussed above emphasize the recharging quality and aim to guarantee that each sensor can be fully recharged. Studies [10,11,12] aim to recharge sensors by adding an energy harvesting system to each sensor. However, the power supplied by these systems, such as solar, wind and thermal, is unstable and unpredictable. On the other hand, in the energy recharging mechanisms proposed in [13,14,15,16,18,19], the recharging paths for mobile rechargers must pass through the central location of each sensor, leading to energy inefficiency. This paper proposed a recharging path reduction mechanism (RPC) which analyzes and constructs the shortest recharging segment for each sensor and ensures the sensor can be fully recharged when the mobile recharger moves along this segment. Compared with the existing works, the proposed RPC reduces the path length and guarantees each sensor to be fully recharged. Table 1 summaries the comparisons of the related researches and the proposed RPC.

## 3. Network Environment and Problem Formulation

This section initially introduces the network environment and the assumptions of this work. Then, the problem formulation is proposed. Subsequently, the sensor recharging model is presented.

### 3.1. Network Environment

Assume that the working scenario of the proposed recharging algorithm is an indoor sensor network. In this scenario, there is no sunlight or other environmental energy recharging mechanisms that can be applied to recharge the sensors. Given a monitoring region *O*, this paper assumes that a set of *h* static sensors $S=\left\{s_{1},s_{2},s_{3},\ldots ,s_{h}\right\}$ is distributed over region *O*, where $s_{1}$ denotes the sink node. All sensors have the same sensing rate and their readings are directly transmitted to the mobile recharger. Therefore, the energy consumption rates of all static sensors are equal. Each static sensor is equipped with a rechargeable battery with limited capacity. A mobile recharger, denoted by *M*, should move with a constant velocity and periodically visit each static sensor, aiming at collecting data from each static sensor and recharging the visited sensor during a predefined period *T*.

**Figure 1:**
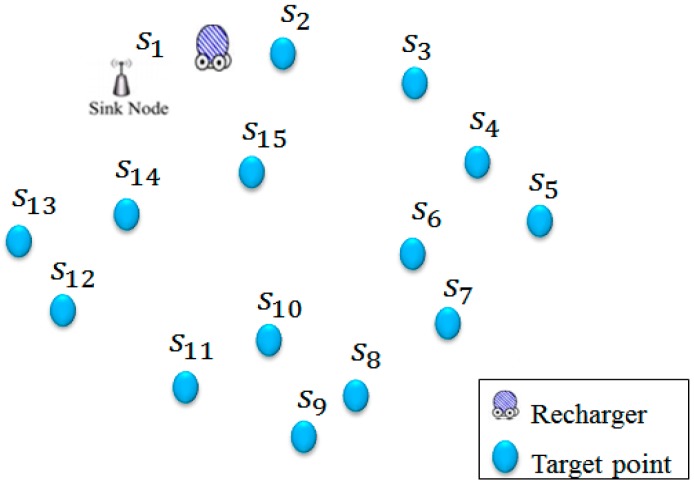
The scenario of the considered WSNs.

Constructing an efficient recharging path for recharger *M* has several challenges. First of all, the shortest path that passes through all sensors is not a good solution. The major reason is that the path might too long since it is not necessary to pass the location of each sensor. In fact, the path only needs to intersect with the recharging range of each sensor. This guarantees at least that the mobile recharger has an opportunity to recharge the sensor. To further guarantee that the sensor can be fully recharged, the length of recharging time period should be accurately evaluated. Another challenge is that the recharging ranges of many sensors might be intersected with each other. It is difficult to construct the shortest path by considering both the factors of required recharging time period and the overlapped recharging ranges of neighboring sensors. This paper aims to construct the shortest path for *M* such that the energy recharge of each sensor can be satisfied. We assume that the information including the total number and the locations of static sensors are known. Figure 1 presents a scenario where a set $S=\left(s_{1},s_{2},s_{3},\ldots ,s_{14}\right)$ of fourteen static sensors and a mobile recharger have been deployed in the region *O*.

### 3.2. Problem Formulation

This paper aims to construct the shortest recharging path for mobile recharger *M* such that each sensor can be fully recharged. Let P denote the recharging path and len() denote the function that returns length of a path. Equation (1) represents the goal of this paper:

(1) Minimize len(P)

The goal given in Equation (1) should satisfy the following three constraints: the first one is the *Sensor Recharging Constraint*, which asks each sensor to be fully recharged when the mobile recharger visits it. That is, in worst case, the recharging energy obtained by each sensor should be equal to or not less than the capacity of the sensor battery. Let $E^{need}$ denote the battery capacity of each sensor. Let $T_{i}$ denote the time period that mobile recharger falls in the recharging range of sensor $s_{i}$. Let $T_{i}=k_{i}\cdot t_{unit}$=$\left[t_{i}^{1},t_{i}^{k_{i}}\right]$ where notation $t_{unit}$ denotes the length of each time slot and let $e_{i}^{t}$ denote the obtained energy of sensor $s_{i}$ from the mobile recharger *M* at time point *t*. The following *Sensor Recharging Constraint* asks each sensor to be fully recharged:

(2) $$\int_{t_{i}^{1}}^{t_{i}^{k_{i}}}e_{i}^{t}\;\mathrm{dt}\geq E^{need}\;where\;0\leq i\leq h-1$$

Another constraint is required to guarantee that each sensor’s energy can support the energy consumptions for sensing and communication. Recall that the mobile recharger *M* travels along path P takes time period $T_{p}$. That is to say, each sensor can be recharged again every time period $T_{p}$. Equation (3) depicts the *Network Lifetime Constraint*:

(3) $$\left(e^{sen}+e^{com}\right)\cdot \frac{T_{p}}{t_{unit}}\leq E^{need}$$

The third constraint is to guarantee that all sensors that fall in the recharging range of the mobile recharger can obtain the energy from mobile recharger simultaneously. Let $S^{t}$ denote the set of sensors which satisfy the condition that mobile recharger falls in the sensor’s recharge range at time *t*. Let notation $b_{i}^{t}$ be a Boolean variable representing whether or not the sensor $s_{i}$ is recharged at the time *t*. Assume that the number of elements in $S^{t}$ is $k^{t}$. The following *Recharging Neighbors Constraint* should be satisfied:

(4) $$\underset{s_{i}\in S^{t}}{\sum }\;b_{i}^{t}=k^{t}$$

### 3.3. Sensor Recharging Model

Let P denote the path along which the mobile recharger moves and recharges each sensor’s battery. This section aims to analyze the energy obtained by the sensor when the recharger moves along path *P*. Recall that notations *M* and $s_{i}$ denote the recharger and the recharged sensor, respectively. We aim to construct path P along which each sensor can be fully recharged while the length of P can be as short as possible.

The mobile recharger *M* moving along path P and recharging each sensor $s_{i}$ should guarantee that the battery of each sensor is fully recharged. Let d be the distance between *M* and $s_{i}$. It is obvious that the recharged energy of $s_{i}$ is decreased with the distance *d*. Let $P_{M}^{tx}$ denote the recharging power applied by recharger *M*. Let notations $G^{tx}$ and $G^{rx}$ denote the antenna gains of *M* and $s_{i}$, respectively. Let λ denote by the amplitude, $L^{p}$ denote the polarity loss, and $P_{s_{i}}^{rx}$ denote the power received by $s_{i}$. According to Friis’s free space equation [17], the recharging power obtained by $s_{i}$ from a fix recharger *M* can be formulated as Equation (5):

(5) $$P_{s_{i}}^{rx}=\frac{G^{rx}G^{rx}\eta }{L^{p}}{\left(\frac{\lambda }{4\pi \left(d+\beta \right)}\right)}^{2}P_{M}^{tx}$$

where η is referred to as rectifier efficiency, and β is a parameter to adjust the Friis transmission equation to room environment.

According to Equation (5), the distance d is an important parameter in recharging model. A large value of d will lead to low recharging efficiency while a small value of d might result in a long path. Let notation $r^{rch}$ represent the threshold of distance. It is obvious that we have:

(6) $$R_{i}=\pi \cdot {\left(r^{rch}\right)}^{2}$$

when condition $r^{rch}\leq d$ is satisfied, the recharging energy obtained by $s_{i}$ can be neglected. That is, $P_{s_{i}}^{rx}=0$. Let the location of $s_{i}$ be (0, 0). The battery energy of $s_{i}$ received from *M* which is located at position (*x*, *y*) is represented as Equation (7):

(7) $$P_{s_{i}}^{rx}\left(x,y\right)=\{\begin{array}{c} \frac{\tau }{{\left(d+\beta \right)}^{2}},\;d\leq r^{rcg}\\ 0,\;d\geq r^{rcg} \end{array}$$

where $\tau =\frac{G^{tx}\times G^{rx}\times \eta }{L^{p}}\times {\left(\frac{\lambda }{4\times \pi }\right)}^{2}\times P_{M}^{tx},$ and $d=\sqrt{x^{2}+y^{2}}$.

## 4. Recharging Path Construction (RPC) Algorithm

This section presents the proposed RPC algorithm, which aims to reduce the length of recharging path while satisfying energy recharging requirements of all sensors. The proposed RPC mainly consists of four phases. The first phase aims to construct an initial recharging path that passes through all sensors. Based on the constructed path, all sensors are ordered in a certain sequence. The second phase aims to divide the ordered sensors into many groups. The third phase aims to reduce the length of the subpath of each group. Finally, the fourth phase interconnects the subpaths of all groups and forms the recharging path.

### 4.1. Initial Recharging Path Construction (IRPC) Phase

Recall that the set of sensors is represented by $S=\left\{s_{1},s_{2},\ldots s_{h}\right\}$. Let $\left(x_{i},y_{i}\right)$ denote the location of sensor $s_{i}$. Let $s_{southeast}$ be the southeast point in *S*. That is:

(8) $$s_{southeast}=arg\;\underset{s_{i}\in S}{min}\;y_{i}$$

Initially, the $s_{southeast}$ will be chosen as the first point for constructing the path. The path *P* that passes each $s_{i}\in S$ will be constructed point by point. The *IRPC* *Phase* mainly consists of three steps: the convex polygon construction, the remaining points connection and the renumbering steps. The following describes the first step.

Step 1: Convex Polygon Construction

Initially, let ${\hat{s}}_{1}=s_{southeast}$ will be the first point. Let *φ* be a horizontal line passing through $s_{southeast}$ and *φ* has an infinite length. Then we turn *φ* in a counterclockwise direction until it touches any point, say ${\hat{s}}_{2}$. Then sensor ${\hat{s}}_{2}$ will play the role of $s_{southeast}$ and repeatedly executes the operations describe above to find the next point ${\hat{s}}_{3}$. Let ${\hat{s}}_{k}$ be the last point which find ${\hat{s}}_{1}$ as its next point. Then we have constructed a path $P^{init}=\left({\hat{s}}_{1},\;{\hat{s}}_{2},\ldots {\hat{s}}_{k}\right)$ which forms convex polygon *G*.

Step 2: Remaining Points Connection

This step will be executed if k<h. This implies that there should be h−k+1 remaining points that are inside *G* but are not included in $P^{init}$. In this step, the remaining h−k+1 points should be included in the polygon. Let $S^{rem}$ be the set of remaining h−k+1 points. The point in $S^{rem}$ that is closest to path $P^{init}$ will be chosen as first point by applying Equation (9):

(9) $$s_{closest}=\arg\underset{s_{j}\in S^{rem},{\hat{s}}_{i}\in G}{\min}\left[dist\left(s_{j},{\hat{s}}_{i}\right)+dist\left(s_{j},{\hat{s}}_{i+1}\right)-dist\left({\hat{s}}_{i},{\hat{s}}_{i+1}\right)\right]$$

Then the point $s_{closest}$ will be included in polygon *G* to form a new polygon by connecting $s_{j}$ to two points ${\hat{s}}_{i}$ and ${\hat{s}}_{i+1}$ and removing the edge of ($s_{i}$, $s_{i+1}$). The above mentioned operations will be applied repeatedly until all h−k+1 points have been included in the polygon. 

Step 3: Renumbering

Let the constructed polygon be $G=\left({\hat{s}}_{1},\ldots ,\;{\hat{s}}_{i},s_{j},\ldots ,{\hat{s}}_{k}\right)$. Let the sink node be the *x*th point in *G*. In this step, the sensors in *G* will be renumbered such that the sink node will be the first point in the constructing path. Therefore, the renumbered path will be:

(10) $$P_{I}^{init}=\left({\hat{s}}_{x},{\hat{s}}_{\left(x+1\right)mod\;h},\ldots ,{\hat{s}}_{\left(x+h-1\right)mod\;h}\right)$$

The renumbered path *P* which starts from sink node can be represented as $P_{I}^{init}=\left({\tilde{s}}_{1},{\tilde{s}}_{2},{\tilde{s}}_{3},\ldots ,{\tilde{s}}_{h}\right)$ where ${\tilde{s}}_{i}={\hat{s}}_{\left(x+i-1\right)mod\;h}$.

**Figure 2:**
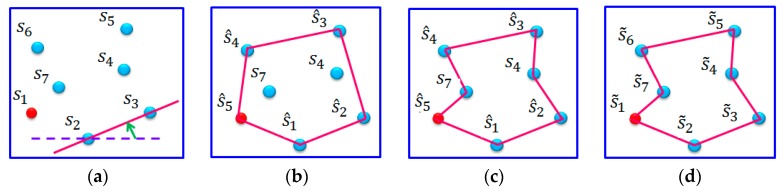
An example of executing the Initial Recharging Path Construction Phase. (**a**) First node and path construction direction of RPC (**b**) The initial path by convex polygon construction (**c**) After execute the remaining points connection (**d**) Renumbering all the connected sensor’s name.

The following gives an example of the proposed *IRPC* *Phase*. Figure 2 depicts the set of seven sensors $S=\left\{s_{1},s_{2},\ldots s_{7}\right\}$. Herein, the sink node is also considered as a sensor, and is denoted by $s_{1}$.

In the first step of this phase, a convex polygon must be constructed starting at the sensor $s_{southeast}=s_{2}$. Let ${\hat{s}}_{1}=s_{2}$. As shown in Figure 2a, the dotted horizontal line is *φ.* Then, turn *φ* in a counterclockwise direction until it touches the first point $s_{3}$, which plays the role of ${\hat{s}}_{2}$. Similarly, treating sensor ${\hat{s}}_{2}$ as $s_{southeast}$, the point ${\hat{s}}_{3}$ can be identified. Repeatedly perform the abovementioned operations, until ${\hat{s}}_{1}$ is finally identified. Then the path $P^{init}$ can be constructed as:

$$P^{init}=({\hat{s}}_{1}=s_{2},\;{\hat{s}}_{2}=s_{3},\ldots {\hat{s}}_{5}=s_{1})$$

As shown in Figure 2b, the convex polygon *G* is obtained.

The second step aims to add the remaining points to convex polygon *G*. If all the sensors have been included in *G*, this step can be ignored. On the contrary, the second step should be applied. In this example, the set of remaining points are $S^{rem}=\{s_{4}$, $s_{7}\}.$ According to Equation (13), sensors $s_{4}$ and $s_{7}$ should be further added to *G*, as shown in Figure 2c. Let $P_{I}^{RPC}$ denoted by the initial recharging path. By applying the third step, the sensors in *S* can be renumbered as $P_{I}^{RPC}=\left({\tilde{s}}_{1},{\tilde{s}}_{2},{\tilde{s}}_{3},\ldots ,{\tilde{s}}_{7}\right)$, as shown in Figure 2d.

After finishing the *IRPC Phase*, an initial path that passes each sensor has been constructed. However, the path length might too long. To reduce the path length of $P_{I}^{RPC}$, the next phase of the proposed *RPC* will simply partition the ordered sensors into groups.

### 4.2. Partitioning Phase

According to the path constructed in the first phase, all sensors are well ordered. This phase aims to partition the ordered sensors into several groups. The motivation of the partitioning task is to simplify the path reduction design. In the later phases, the path reduction will be performed group by group and then the reduced subpaths of all groups will be interconnected as a recharging path.

In fact, the path reduction is a big challenge. Each sensor $s_{i}$ can have many neighbors. It is difficult for determining the previous and next visited sensors by selecting sensors from neighboring sensors of $s_{i}$. This occurs because that the length of recharging path is highly related to the positional relationship of two adjacent sensors. To simplify the path reduction, this phase partitions all sensors into groups.

The partitioning phase will construct three partitions $C_{1}$, $C_{2}$ and $C_{3}$, regardless the number of sensors. Each partition consists of ⌈h/3⌉ disjoint groups, and each group contains exactly three sensors. Since three sensors can form a triangle, the property that the sum of lengths of two edges must larger than the length of the remaining edge. Based on this property, the length reduction operation can be applied to reduce the subpath of each group. The following formally list the three partitions. Each partition $C_{i}$ will be the input of later phases, aiming to construct the reduced recharging path:

$$C_{1}=\{g_{1}^{i}|\;g_{1}^{i}=\left(s_{3*\left(i-1\right)+1},s_{3*\left(i-1\right)+2},s_{3*\left(i-1\right)+3}\right),1\leq i\leq \left\lceil h/3\right\rceil \}$$

$$C_{2}=\{g_{2}^{i}|\;g_{2}^{i}=\left(s_{3*\left(i-1\right)+2},s_{3*\left(i-1\right)+3},s_{3*\left(i-1\right)+4}\right),1\leq i\leq \left\lceil h/3\right\rceil \}$$

$$C_{3}=\{g_{3}^{i}|\;g_{3}^{i}=\left(s_{3*\left(i-1\right)+3},s_{3*\left(i-1\right)+4},s_{3*\left(i-1\right)+5}\right),1\leq i\leq \left\lceil h/3\right\rceil \}$$

**Figure 3:**
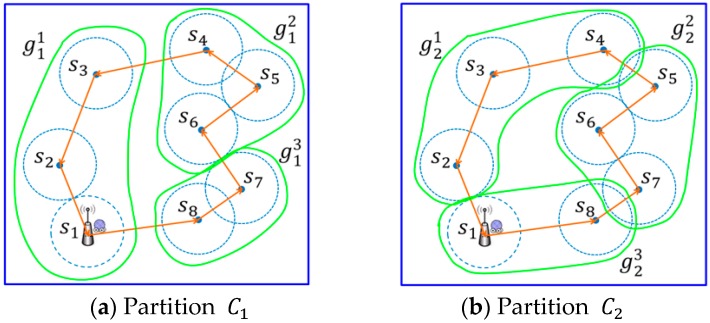
An example of two partitions for path $P_{I}^{RPC}$.

Take Figure 3 as an example. Based on the output of IRPC phase, we have path $P_{I}^{RPC}=\left\{s_{1},s_{2},\ldots s_{8}\right\}$. Partitioning Phase will create the following three partitions:

$$C_{1}=\{\left\{g_{1}^{1}=\left(s_{1},s_{2},s_{3}\right)\right\},\left\{g_{1}^{2}=\left(s_{4},s_{5},s_{6}\right)\right\},\left\{g_{1}^{3}=\left\{\left(s_{7},s_{8}\right)\right\}\right\}$$

$$C_{2}=\left\{\left\{g_{2}^{1}=\left(s_{2},s_{3},s_{4}\right)\right\},\left\{g_{2}^{2}=\left(s_{5},s_{6},s_{7}\right)\right\},\left\{g_{2}^{3}=\left(s_{8},s_{1}\right)\right\}\right\}$$

$$C_{3}=\left\{\left\{g_{3}^{1}=\left(s_{3},s_{4},s_{5}\right)\right\},\left\{g_{3}^{2}=\left(s_{6},s_{7},s_{8}\right)\right\},\left\{g_{3}^{3}=\left(s_{1},s_{2}\right)\right\}\right\}$$

### 4.3. Inner-Group Path Reduction Phase

This phase aims to reduce the subpath for each group. The path reduction scheme consists of two major tasks. The major work of this phase is to construct a chord as the recharging segment for each sensor. To achieve this, two tasks should be performed in this phase. The first task aims to analyze the length of the recharging segment. Then the second task further constructs the recharging segment for each sensor and connects the segments of sensors in each group. The constructed recharging segment should support the property of ‘recharging while moving’. That is, the mobile recharger moves along the constructed segment can fully recharge the battery of that sensor. The following analyzes the length of the recharging segment.

Step 1: Analyzing the Length of the Recharging Segment

It is obvious that the static sensor can be recharged only if the mobile recharger *M* is within *R_i_*. The following presents how to construct a recharging segment for each sensor. Recall that the segment $l_{i}=\left(p_{i},q_{i}\right)$ is a straight line between entering point $p_{i}$ and leaving point $q_{i}$ and $len\left(l_{i}\right)$ denote the length of $l_{i}$. The total recharging energy of $s_{i}$ obtained from the *M* can be evaluated by applying Equation (11):

(11) $$E^{H}=\int_{0}^{\frac{len\left(l_{i}\right)}{v}}\frac{\tau }{{\left(v\times t+\beta \right)}^{2}}\;dt=\frac{\tau \times len\left(l_{i}\right)}{v\times \beta \times \left(len\left(l_{i}\right)/2+\beta \right)}$$

**Figure 4:**
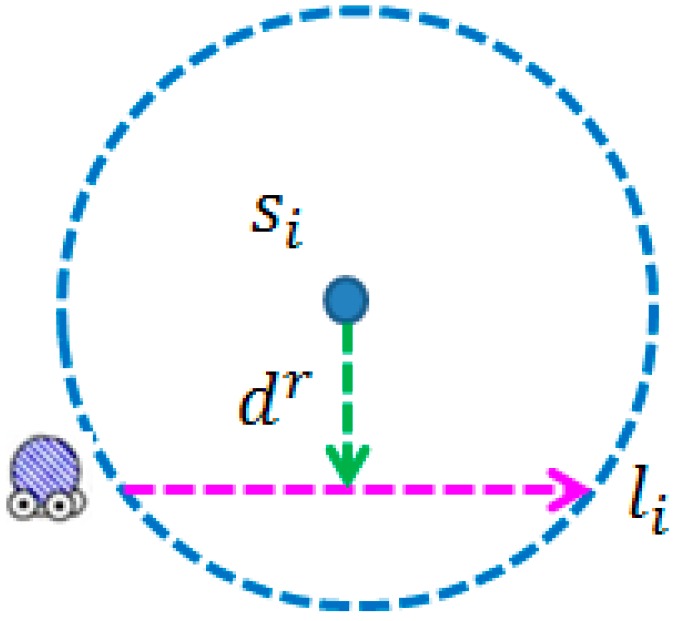
The recharging segment of sensor $s_{i}$.

There are infinite recharging segments in each recharging range. This paper aims to reduce the length of recharging segment $l_{i}$ for each sensor while satisfying the *Sensor Recharging (SR) Constraint* (1). Recall that each sensor has the same battery capacity $E^{need}$. As shown in Figure 4, the constructed recharging segment $l_{i}$ should satisfy the constraint (1).

That is, the sensor can obtain at least recharged energy $E^{need}$ after *M* completing the movement of segment $l_{i}$. Let notation $d^{r}$ denote the shortest distance from point $s_{i}$ to $l_{i}$. Equation (12) reflects the fact mentioned above:

(12) $$2\int_{0}^{\frac{len\left(l_{i}\right)}{2v}}\frac{\tau }{\sqrt{{\left(v\times t\right)}^{2}+{\left(d^{r}\right)}^{2}}+\beta }\;dt-E^{need}\geq 0$$

where $d^{r}\leq r^{rch}$.

According to Equation (12), the lengths of $l_{i}$ and $d^{r}$ can be calculated. The distance from sensor $s_{i}$ to the recharging segment $l_{i}$ should be equal or less then $d^{r}$, in order to guarantee the full recharge of sensor $s_{i}$. In addition to the first task, the next task aims to further construct the recharging segment and the reduced subpath for each group.

Step 2: Constructing the Reduced Subpath for Each Group

This task aims to reduce the length of subpath for each group. The major work of this task is to construct the recharging segment $l_{i}$ for middle sensor of each group. By applying the Triangle Theorem, a reduced recharging path of a group can be constructed. Then, we apply the proposed operations to each group of three partitions $C_{1}$, $C_{2}$ and $C_{3}$. As a result, the reduced subpath of each group can be constructed.

**Figure 5:**
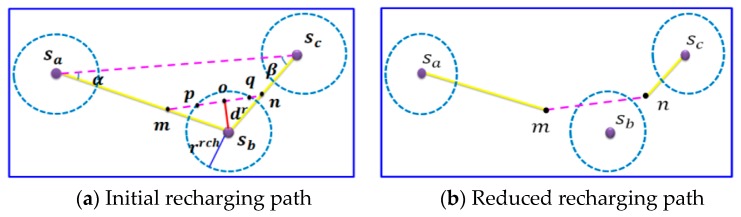
An example of executing Inner-Group Path Reduction Phase.

Assume that there is a group $g_{i}^{j}$ which contains three sensors $s_{a}$, $s_{b}$ and $s_{c}$. The goal of this step is to reduce the recharging path that connects $s_{a}$, $s_{b}$ and $s_{c}$. The following uses Figure 5 as an example to illustrate how to reduce the length of the recharging path.

As shown in Figure 5a, the yellow path represents the initial recharging path of group $g_{i}^{j}$. Let $s_{b}$ be the middle sensor of group $g_{i}^{j}$, the length that falls inside the recharging range of sensor $s_{b}$ is $2r^{rch}$. The path reduction can be achieved by the following operations. The triangle connecting sensors $s_{a},s_{b}\;\mathrm{and}\;s_{c}$ is denoted by $\Delta s_{a}s_{b}s_{c}$, as shown in Figure 5a. We shift the segment $l_{ac}$ toward $s_{b}$, until the distance between sensor $s_{b}$ and the segment $l_{ac}$ is equal to $d^{r}$. Let points p and q represent the intersecting points of recharging circle of sensor $s_{b}$ and segment $l_{ac}$. The path $l_{pq}$ in this example can be considered as the $l_{i}$, which has the important property that the recharger *M* moving along the recharging segment $l_{pq}$ with a constant velocity v can fully recharge the battery of sensor $s_{b}$. As a result, a new recharging path of group $g_{i}^{j}=\left\{s_{a},s_{b},s_{c}\right\}$ can be constructed, as shown in Figure 5b. Compared with the initial path in Figure 5a, the length of the constructed path in Figure 5b has been significantly reduced. The above mentioned operations should be applied to all groups such that the subpath length for each group can be reduced.

### 4.4. Inter-Group Path Reduction Phase

The previous phase has reduced the subpath for each group. This phase aims to further interconnect the subpaths of all groups, forming the reduced recharging path. The major work in this phase mainly consists of two steps. The first step aims to construct the new recharging path of each partition. The second step calculates the saving path length of each partition, and then considers the shortest reduced recharging path as the final recharging path. 

Step 1: New recharging path construction for each partition

**Figure 6:**
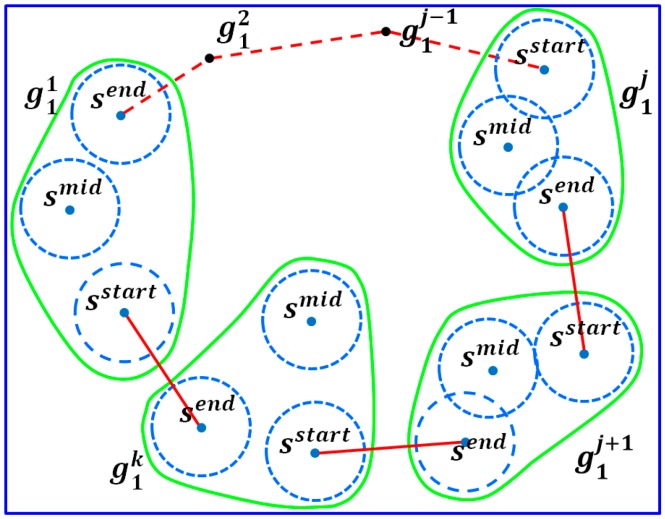
New recharging path of a partition.

This step aims to connect all the subpaths constructed in Phase 3 such that a path for the considered partition can be formed. Consider a particular partition $C_{i}\left(i=1,2,3\right)$ which consists of k groups $g_{i}^{1},g_{i}^{2},\ldots ,g_{i}^{\left\lceil h/3\right\rceil }$, where k=1,…,⌈h/3⌉ and $g_{i}^{j}$ is in the previous order of $g_{i}^{j+1}$ along the clockwise direction. In this example we connect the reduced subpath starting from the group $g_{i}^{j}$. Let $L_{i}^{j}$ represent the reduced recharging path of group $g_{i}^{j}$. Let notations $s_{\left(i,j\right)}^{start}$ and $s_{\left(i,j\right)}^{end}$ denote the starting and ending sensors of path $L_{i}^{j}$, respectively. By connecting the ending sensor $s_{\left(i,j\right)}^{end}$ in group $g_{i}^{\left(j\;mod\;k\right)}$ to the starting sensor $s_{\left(i,j+1\right)}^{start}$ in group $g_{i}^{\left(j+1\right)\;mod\;k}$, 1≤j≤k, a final path for a certain partition can be obtained. Figure 6 gives an example of a new reduced recharging path of a partition.

**Figure 7:**
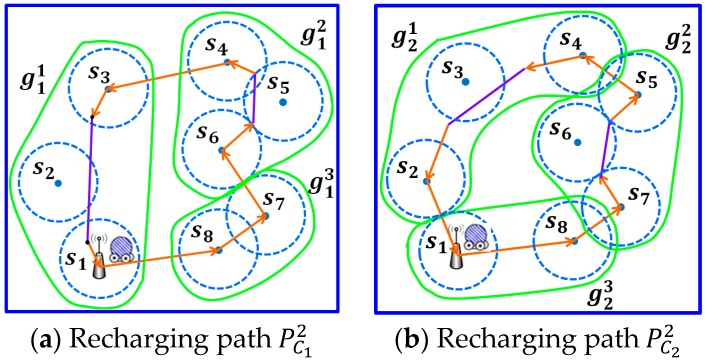
The reduction path of each partition by applying Step 1 on the example in Figure 3.

By applying the operations proposed in this step to each partition of $C_{1},C_{2}$ and $C_{3}$, three new reduction paths can be constructed. As shown in Figure 7, there are eight sensors in the monitoring area. Let notations $P_{C_{1}}^{1}$ and $P_{C_{2}}^{1}$ represent the initial paths of $C_{1}$ and $C_{2}$, respectively, while notations $P_{C_{1}}^{2}$ and $P_{C_{2}}^{2}$ denote the reduced path of $C_{1}$ and $C_{2}$, respectively.

Step 2: The Selection of Recharging Path

This step aims to choose the shortest recharging path from the constructed paths in the first step. As discussed above, three new recharging paths $P_{C_{i}}^{2}\left(i=1,2,3\right)$ have been constructed. Then we calculate the length of path $P_{C_{1}}^{1}$, $P_{C_{2}}^{1}$ and $P_{C_{3}}^{1}$.

Take Figure 5 as an example. Let notations α and β denote the angles of $∠s_{b}s_{a}s_{c}$ and $∠s_{b}s_{c}s_{a}$, respectively, as shown in Figure 5a. Let $w_{ij}$ denote the saving length of recharging path of group $g_{i}^{j}$. Compared with Figure 5a, the saving path length in Figure 5b can be represented as Equation (13):

(13) $$w_{i,j}=l_{s_{b}m}+l_{s_{b}n}-l_{mn}$$

As shown in Figure 5a, the lengths of segments $l_{om}$ and $l_{on}$ could be expressed by Equations (14) and (15), respectively, according to Pythagorean theorem:

(14) $$l_{om}=\frac{r^{rch}}{tan\beta }$$

(15) $$l_{on}=\frac{r^{rch}}{tan\alpha }$$

Based on Equations (14) and (15), we have:

(16) $$l_{mn}=l_{om}+l_{on}=\frac{r^{rch}}{tan\beta }+\frac{r^{rch}}{tan\alpha }$$

Similarly, segments $l_{bm}$ and $l_{bn}$ can be calculated by applying Equations (17) and (18), respectively:

(17) $$l_{bn}=\frac{r^{rch}}{sin\alpha }$$

(18) $$l_{bm}=\frac{r^{rch}}{sin\beta }$$

Substituting Equations (16)–(18) into Equation (13), we have:

(19) $$w_{i,j}=l_{bm}+l_{bn}-l_{mn}=\frac{r^{rch}}{sin\beta }+\frac{r^{rch}}{sin\alpha }-\left(\frac{r^{rch}}{tan\beta }+\frac{r^{rch}}{tan\alpha }\right)$$

Let $C_{i}^{w_{ij}}\left(1\leq i\leq 3\right)$ denote the total saving path length of partitions $c_{i},\left(1\leq i\leq 3\right)$. The values of $C_{1}^{w_{ij}}$, $C_{2}^{w_{ij}}$ and $C_{3}^{w_{ij}}$ can be obtained by applying Equation (20):

(20) $$C_{i}^{w_{ij}}=\overset{j=\left\lceil h/3\right\rceil }{\underset{i,j=1}{\sum }}w_{ij},\;1\leq i\leq 3$$

where variable i denotes the sequence number of partitions while variable j denotes the sequence number of group in partition $C_{i}$. Then, the partitions which has maximal saving length will be considered as the best recharging path, called $P^{best}$.

### 4.5. The Proposed RPC Algorithm

This subsection presents the *RPC* algorithm by summarizing the operations presented previous subsections of Section 4. Algorithm 1 depicts the detailed steps designed for the proposed RPC algorithm. In Phase I, steps 1–8 select the southeast sensor as an initial node to construct a convex polygon *G*. Next, if k<n, steps 9–15 connect the remaining points to *G*. In Phase II, steps 16–18 divide all sensors into different groups, and three partitions $C_{1},C_{2}$ and $C_{3}$ can be formed. In Phase III, for a group $g_{i}^{j}$, steps 19–29 construct a recharging segment $l_{mn}$ to replace the subpaths $l_{mk}$ and $l_{kn}$. Then, in the Phase IV, steps 24–26 calculate the value of $w_{ij}$ for each group. Steps 27–29 calculate the saving length of partitions $C_{i}$, and select the shortest path as the final recharging path, called $P^{best}$.
**Algorithm 1: Recharging Path Construction (RPC) Algorithm**
Inputs: 1. A set of sensors $S=\left\{s_{1},s_{2},\ldots ,s_{h}\right\}$. Notation$\left(x_{i},y_{i}\right)$ denotes the location of sensor $s_{i}$. The mobile recharger is labeled with $s_{1}$. 2. The southeast point $s_{southeast}$, horizontal line *L* passing through $s_{southeast}$.Output: The recharging path $P^{best}$.P H A S E I/* Initial Recharging Path Construction (*IRPC*) Phase */1.  for(i=1,i≤h,i++){2.   $s_{southeast}=arg\underset{s_{i}\in S}{\min}\left(y_{i}\right);$
3.  ${\hat{s}}_{1}=s_{southeast}$
4.  Turn *L* in an anticlockwise direction until *L*5.  touches any point, say ${\hat{s}}_{i+1}$;6.  ${\hat{s}}_{i+1}$ plays the role of $s_{southeast}$;7.  goto 4;8.  Connect each ${\hat{s}}_{i+1}$;}9.  Let convex polygon *G*=${\hat{s}}_{1},\;{\hat{s}}_{2},\ldots {\hat{s}}_{k}$;10.  if (k<n){11.  $S^{rem}=s_{1},\ldots s_{n-k+1}$;12.  for(j=1,j≤n−k,j++){13.  compute $s_{closest}$ according to Exp. (10)14.  $l_{s_{i},\;s_{i+1}}\leftarrow l_{s_{i},\;s_{j}}+l_{s_{j},\;s_{i+1}}$;}}15.  $\mathrm{Let}\;P=\left({\tilde{s}}_{1},{\tilde{s}}_{2},{\tilde{s}}_{3},\ldots ,{\tilde{s}}_{n},\right)$;P H A S E II/*Partitioning Phase*/16.  for(j=1,j≤h/3,j++){17.  $g^{j}$=($s_{i},s_{i+1},s_{i+2}$);18.  Construct three partitions $C_{1},C_{2}$ and $C_{3}$};P H A S E III/*Inner-Group Path Reduction Phase*/19.  for(j=1,j≤h/3,j++){20.  $g_{i}^{j}=\left(s_{k-1},s_{k},s_{k+1}\right)$;21.  Construct $\Delta s_{k-1}s_{k}s_{k+1}$;22.  Shift the segment $l_{\left(k-1\right)\left(k+1\right)}$ toward $s_{k}$;23.  $l_{mk}+l_{kn}\leftarrow l_{mn}$;P H A S E IV/*Inter-Group Path Reduction Phase*/24.  for(j=1,j≤h/3,j++){25.  for(i=1,i≤3,i++)26.  Compute $w_{i,j}$ according to Exp. (19);}27.  Compute $C_{1i}^{w_{ij}}$, $C_{2}^{w_{ij}}$ and $C_{3}^{w_{ij}}$ by Exp. (20);28.  $P^{best}=\min\left(C_{1}^{w_{ij}},C_{2}^{w_{ij}},C_{3}^{w_{ij}}\right)$;29.  Return $P^{best}$;

## 5. Performance Evaluation

This section presents the performance evaluation of the proposed RPC method in terms of recharging path length and energy efficiency. The proposed RPC algorithm is compared with the HAM-based recharging mechanism [18], the approach proposed in [19], which is referred to OWER-MDG and the optimal recharging path. The HAM-based recharging mechanism mainly applies the Hamiltonian algorithm to construct the recharging path which passes through the center of each sensor. The detailed scheme of the OWER-MDG mechanism has been reviewed in the related work of this paper.

**Figure 8:**
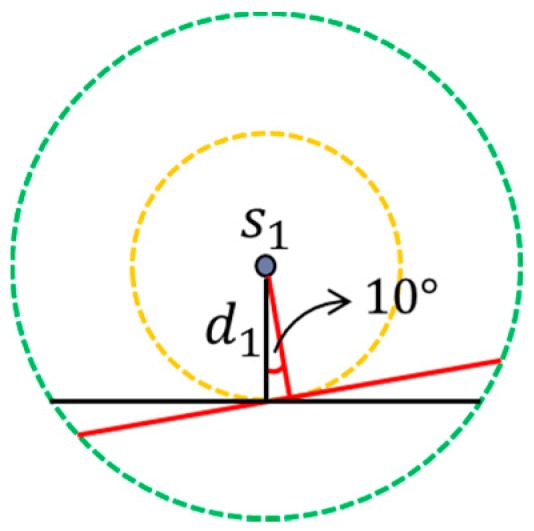
The recharging segments of a sensor.

**Figure 9:**
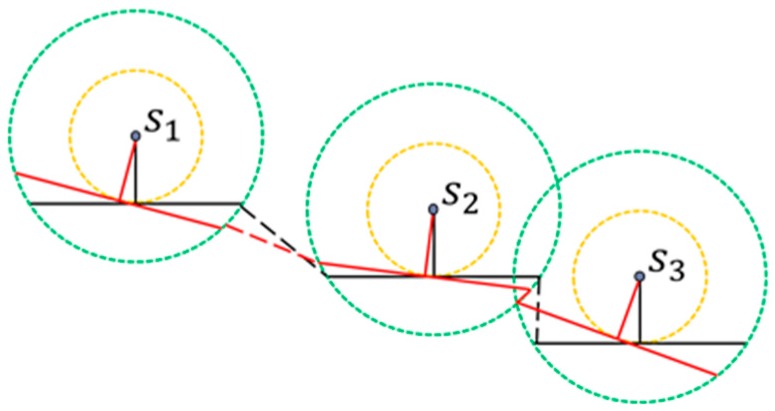
An example that applies the exhausted search to obtain the near optimal mechanism.

To investigate how well the proposed RPC algorithm performs, we should compare the proposed RPC with the optimal result, but to our knowledge, there is no optimal mechanism proposed in literature for recharging paths. The “recharging while moving” can effectively reduce the total length of the path of mobile recharger, as compared with the most existing mechanisms that the recharging path passes the location of each sensor. Therefore, we propose a near optimal mechanism which applies exhausted search to find the near optimal path. To satisfy the three constraints given in problem formulation section, the mobile recharger should move along the recharging segment of each sensor. However, there are infinite numbers of recharging segments in a sensor. The best recharging segment depends on the relative locations of neighboring sensors. To obtain the optimal result, we turn the recharging segment of each sensor every 10 degree, as shown in Figure 8. Then the concept of exhausted search is applied such that all combinations for connecting neighboring recharging segments are considered. The path with shortest length will be treated as the near optimal mechanism and is compared with the proposed mechanism. Figure 9 gives one possible combination of the recharging segments of the neighboring nodes. As shown in Figure 9, the paths marked with black and red inks represent the original and the optimal paths, respectively.

### 5.1. Simulation Environment

**Figure 10:**
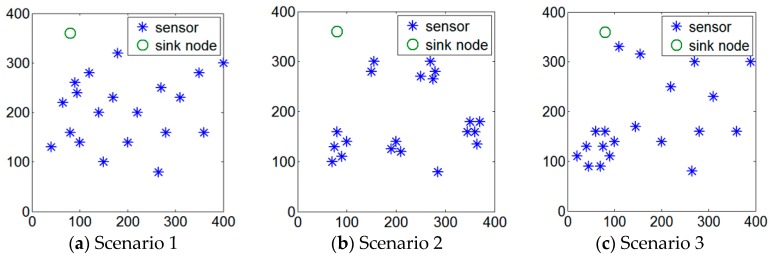
Three scenarios considered in the experiments.

In the experimental study, we use MATLAB 2015 as the simulation tool. The following illustrates the parameters considered in the simulation environment. A set of static sensors are randomly deployed in a given area *O* sized 400 m × 400 m. The number of sensors deployed in area *O* is ranging from 5 to 30. All results are obtained from the average of 100 experiments. Three scenarios of sensor deployments are considered in the experiment, including distributed, centralized as well as their combination. In the distributed scenario, all sensors are randomly deployed over the area *O*. In each round, one location is randomly determined in the area *O* and one sensor is deployed at that location. This operation will be repeated performed until the predefined number of sensors have been deployed. Figure 10a depicts the deployment snapshot of 20 sensors in the distributed scenario.

In the second scenario (centralized scenario), a predetermined number of sensors are randomly partitioned into six groups. There are six locations randomly determined in area *O*. One sensor will be selected from each group and there are totally six sensors selected from six groups. These sensors are called initial sensors which will be deployed at the six determined locations. Then the sensors in the same group will be deployed at the neighborhood location which is randomly determined within the range of 30 units distance far from the initial sensor of the same group. Therefore, all sensors in a group can be closed to each other. Figure 10b depicts the deployment snapshot of 20 sensors using the centralized scenario. The 20 sensors are randomly partitioned into six groups which contain 5, 5, 4, 3, 2, 1 sensors. Then one sensor in each group will be randomly deployed in area *O*. After that, all the other sensors in the same group can be deployed accordingly.

The third scenario is the combination scenario, which combines the abovementioned two scenarios. Initially, one random number ranging from 1 to 10 is generated as the number of sensors in the first group. All the other sensors will be treated as different groups. Each of these groups exactly has one sensor. As shown in Figure 10c, the random number 9 is generated. This indicates that the first group contains nine sensors. All the other 11 sensors are individual groups. Each group contains one sensor. Similar to the first scenario, we randomly determine 10 locations in area *O* and one sensor selected from each group plays the initial sensor and is deployed at the each determined location. After that, similar to the second scenario, the sensors in the same group will be deployed in the neighborhood locations of the initial sensor.

In each scenario, since the location of each sensor has been known, the proposed algorithm will select the southeast sensor, whose location satisfying condition $s_{southeast}=arg\;\underset{s_{i}\in S}{min}\left(y_{i}\right)$, as the first sensor of path $P^{init}$. This sensor will be denoted by ${\hat{s}}_{1}$. Then the path can be constructed by applying the proposed algorithm presented in Section 4.5.

### 5.2. Performance Study

**Figure 11:**
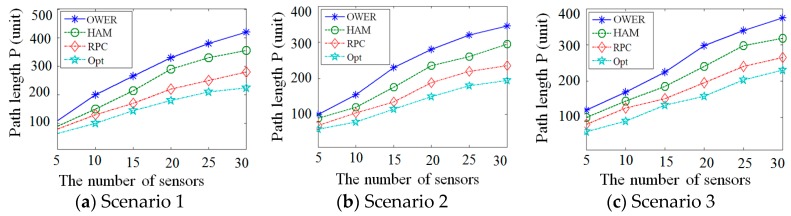
The comparison of four recharging mechanisms in terms of path length using different deployment scenarios.

Figure 11 depicts the recharging path length in three scenarios by applying the four compared algorithms. The number of sensors is ranging from 5 to 30. In general, In Figure 11a–c, the path lengths of four compared algorithms are increased with the number of sensors. This occurs because that the size of area covered by sensors can be enlarged when the number of sensors increased. Therefore, the mobile recharger needs to visit a larger area, leading to the incensement of recharging length. Consider the scenario 1. As shown in Figure 11a, the OWER-MDG mechanism constructs several tours to recharge all sensors in the network. For each recharging tour, SenCar travels along a specific path consisting of some anchor sensors with heavy data traffic. Since the path constructed by OWER-MDG does not visit all sensors in each tour, the recharging path for all sensors can be treated as the connected path of the tours that are constructed by several runs. As a result, the OWER-MDG has longest path. In the HAM-based recharging mechanism, the recharging path is constructed passing through the location of each sensor. Therefore, the recharging path length obtained by applying the HAM-based recharging mechanism is shorter than the OWER-MDG mechanism.

The proposed RPC algorithm derives the recharging segment of each sensor. The mobile recharger travels along a certain chord of recharging range of each sensor. This can guarantee that the sensor can be fully recharged. Then, the proposed RPC algorithm reduces the length of recharging path by executing the Inner-Group Path Reduction Phase and the Inter-Group Path Reduction Phase. As a result, the proposed RPC algorithm constructs a shorter path than the OWER-MDG and HAM-based recharging algorithms in all scenarios, as shown in Figure 11a–c. Since the near optimal mechanism exhaustedly selects the recharging segment of each sensor according to the relative locations of all sensors, it obtains a shorter recharging path, as compared with the other three algorithms.

**Figure 12:**
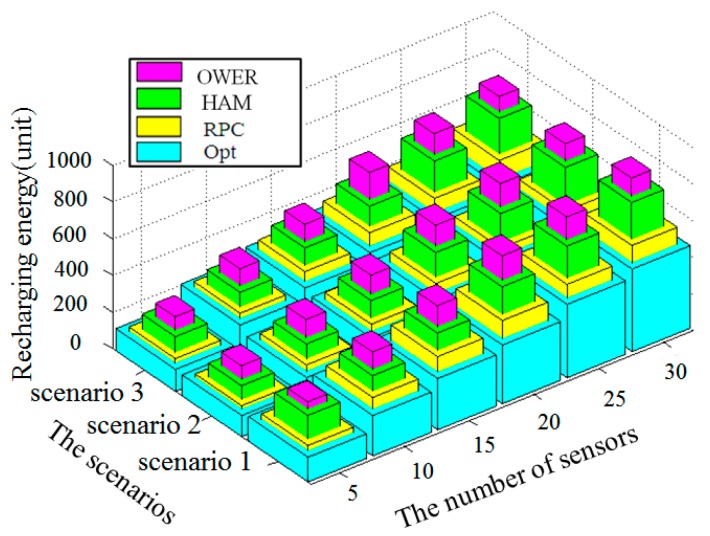
Impact of number of sensors on the energy consumption by applying the four compared algorithms.

The energy consumption of mobile recharger highly depends on the length of recharging path and the number of sensors. The mobile recharger *M* receiving data from a sensor and moving a unit distance consume energy at the rates of 0.075 J/s and 8.267 J/unit [20], respectively. For each static sensor, the energy consumptions for sensing and transmitting data to recharger *M* are set to 0.1 J/s and 0.18 J/s, respectively. In general, the energy consumption of four compared algorithms increased with the number of sensors and length of recharging path. Figure 12 compares the energy consumptions of HAM-based recharging, the OWER-MDG algorithm, the proposed RPC algorithm and the near optimal mechanism, by varying the number of sensors and the adopted three scenarios. The number of sensors varies from 5 to 30 in each scenario. As shown in Figure 12, in each scenario, when the number of sensors increased, the energy consumption is also increased. On the other hand, if we fix the number of sensors, say 20, scenarios 1 and 2 obtain the longest and shortest path lengths, respectively. These results are similar, no matter the applied algorithm is *HAM-based recharging*, *OWER-MDG* algorithm, the proposed *RPC* algorithm or the near optimal mechanism. In comparison, the near optimal mechanism outperforms other three compared algorithms in all cases in terms of energy consumptions. The proposed *RPC* consumes less energy than the *HAM-based recharging* and *OWER-MDG* algorithm in all cases. Therefore, the energy consumption of the proposed *RPC* is closest to the near optimal mechanism.

A good recharging algorithm will create a short recharging path such that the mobile recharger spends less time in travelling the path and spends most of its time recharging the battery of each sensor. The ratio of recharging time to total required time presents the recharging efficiency of a recharging algorithm. The following defines recharging efficiency index, denoted by $I_{time}$, to measure the efficiency of a recharging algorithm. Consider a fixed scenario *x*, where x=a1,a2 and a3 represents the considered scenario 1, scenario 2 and scenario 3, respectively. Let notations $t_{rch}\left(x,y\right)$ and $t_{move}\left(x,y\right)$ denote the recharging time and path traveling time required by algorithm *y*, respectively. The recharging efficiency index, denoted by $I_{time}\left(x,y\right)$, is defined by Equation (21):

(21) $$I_{time}\left(x,y\right)=\frac{t_{rch}\left(x,y\right)}{t_{move}\left(x,y\right)+t_{rch}\left(x,y\right)}$$

**Figure 13:**
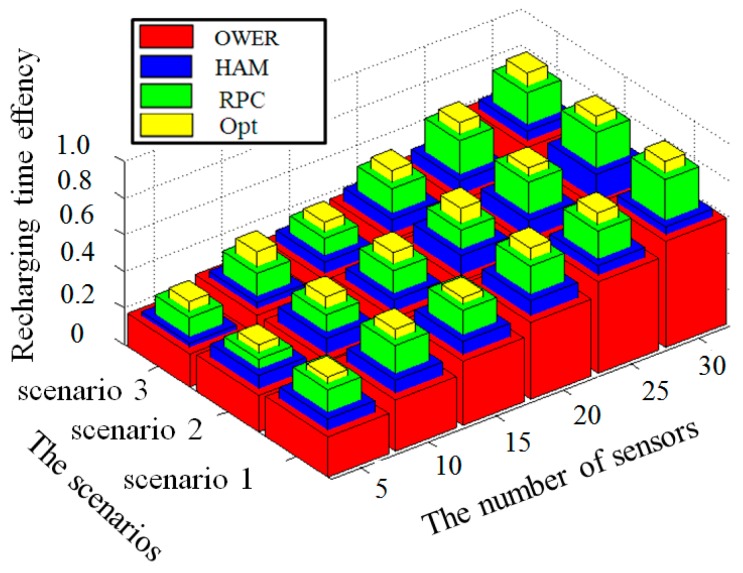
Comparison of the four algorithms in terms of recharging time efficiency in three scenarios.

A larger value of $I_{time}\left(x,y\right)$ indicates the recharging algorithm *y* is more efficient. Figure 13 investigates the recharging efficiency indices by applying the HAM-based recharging, OWER-MDG, the proposed RPC algorithm and the near optimal mechanism in three scenarios.

Consider the HAM-based recharging algorithm. Let notation HAM denote the HAM-based recharging algorithm. Since scenarios 1 and 2 yield the longest and shortest recharging paths, respectively, we have:

$$t_{move}\left(a1,Ham\right)>t_{move}\left(a3,Ham\right)>t_{move}\left(a2,Ham\right)$$

Since the same Hamiltonian algorithm is applied to three scenarios, we have:

$$t_{rch}\left(a1,Ham\right)=t_{rch}\left(a2,Ham\right)=t_{rch}\left(a3,Ham\right)$$

As a result, we have:

$$I_{time}\left(s2,Ham\right)>I_{time}\left(s3,Ham\right)>I_{time}\left(s1,Ham\right)$$

That is, when the applied algorithm is HAM-based recharging algorithm, scenario 2 has the best efficiency. Similarly, no matter whether OWER-MDG or the proposed PRC algorithm are applied, scenario 2 has the best efficiency. The derivations also match the results as shown in Figure 13.

Since the four compared algorithms can similarly achieve the best efficiency in scenario 2, the following discussions use scenario 2 in our experiment environment. In comparison, the near optimal mechanism constructs the shortest travelling path, as compared with the other three mechanisms. The proposed RPC outperforms the OWER-MDG and HAM-based recharging mechanisms in terms of path length. Consequently, we have:

$$I_{time}\left(a2,Opt\right)>I_{time}\left(a2,RPC\right)>I_{time}\left(a2,HAM\right)>I_{time}\left(a2,OWER-MDG\right)$$

**Figure 14:**
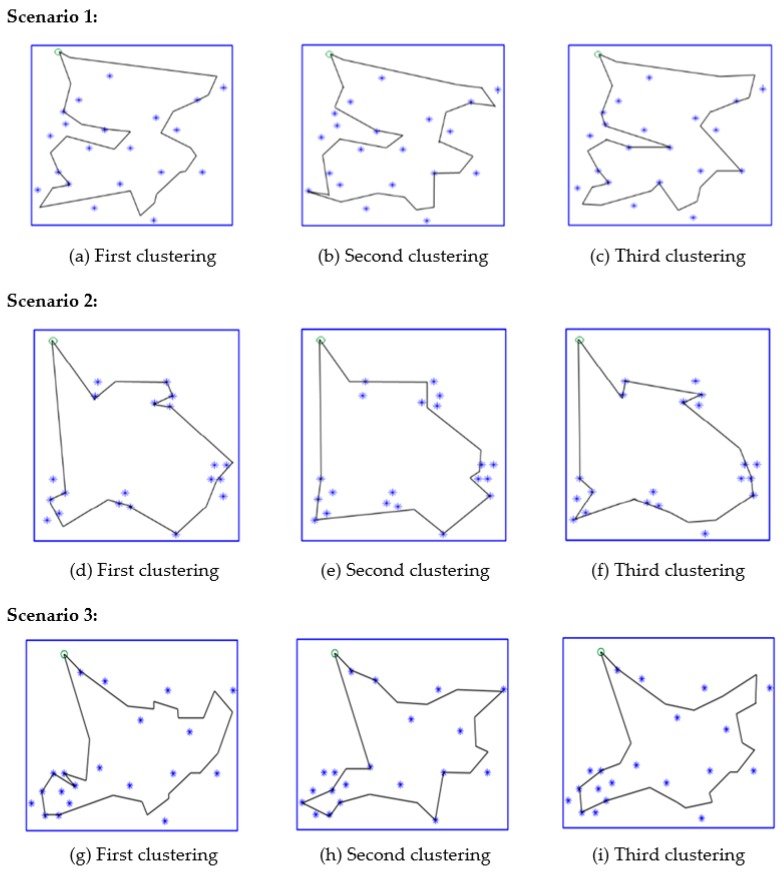
The recharging paths by applying three clustering mechanisms. Three scenarios are considered.

The experiment results shown in Figure 14 also verify the abovementioned discussions.

Recall that there are three clustering mechanisms proposed in the second phase of the proposed RPC algorithm. Figure 14 aims to depict that each of the three clustering mechanisms is possible to obtain the best results, depending on the distributions of the sensor nodes. The proposed RPC algorithm is applied to compares the three clustering mechanisms and selects the best one that can obtain the shortest path. Compare Figure 14a–c. The first clustering mechanism yields the shortest recharging path, as shown in Figure 14a. The second clustering mechanism constructs the longest recharging path.

Scenario 2 has different results. Alternatively, the second clustering mechanism, as shown in Figure 14e, obtains the shortest path. In scenario 3, the proposed RPC chooses the third path, as shown in Figure 14i.

**Figure 15:**
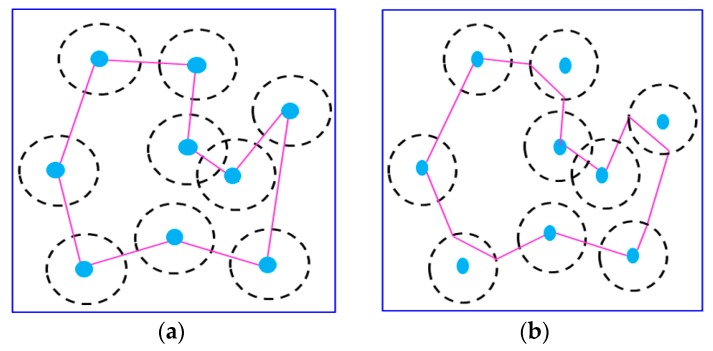
Example of recharging path reduction by applying the second clustering mechanism. (**a**) The path constructed by the First phase of RPC; (**b**) The path constructed by all phases of RPC.

Figure 15 presents an example to show the path reduction using the second clustering as our strategy. The number of sensors is set to 9. Figure 15a represents the recharging path constructed after applying the first phase of the proposed RPC mechanism. As shown in Figure 15a, the recharging path passes through the center of each sensor. Figure 15b shows the recharging path after applying all phases of the proposed RPC mechanism. Obviously, the length of recharging path has been significantly reduced.

**Figure 16:**
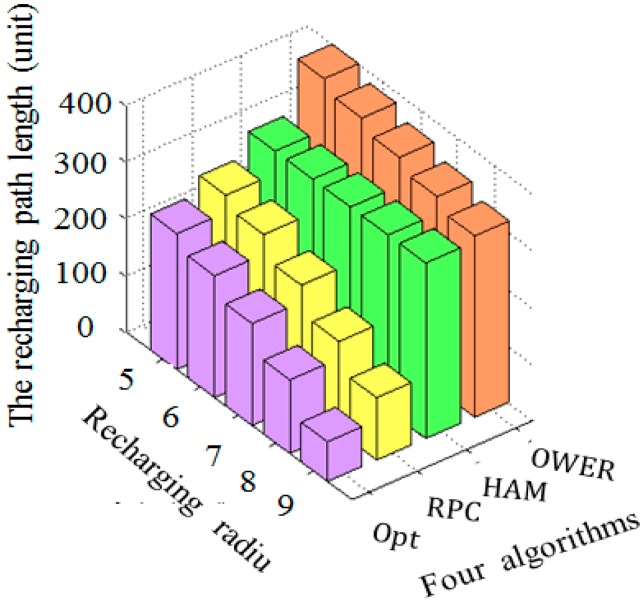
The comparison of path length of the four algorithms by varying the recharging radius ranging from 5 to 9 distance units.

In general, the recharging range of a sensor is an important parameter on the impact of recharging path length. Figure 16 shows the impact of recharging radius on the path length by applying the three compared algorithms. In the Hamiltonian algorithm, the recharging path passes through the center of each sensor, the change of recharging range has no effect on the recharging path length.

As shown in Figure 16, the OWER-MDG, the proposed RPC algorithms and the near optimal mechanism have similar results that the recharging path length is reduced with the recharging radius. This occurs because that the static sensor can obtain the energy from the mobile recharger, even though their distance is long. In comparison, the OWER-MDG algorithm results in longest path since the visiting of all sensors requires several runs. The proposed RPC algorithm constructs a shorter recharging path than the OWER-MDG and HAM-based mechanisms. Since the near optimal mechanism applies an exhaustive search to construct the recharging path, it constructs the shortest path, as compared with the other three algorithms.

**Figure 17:**
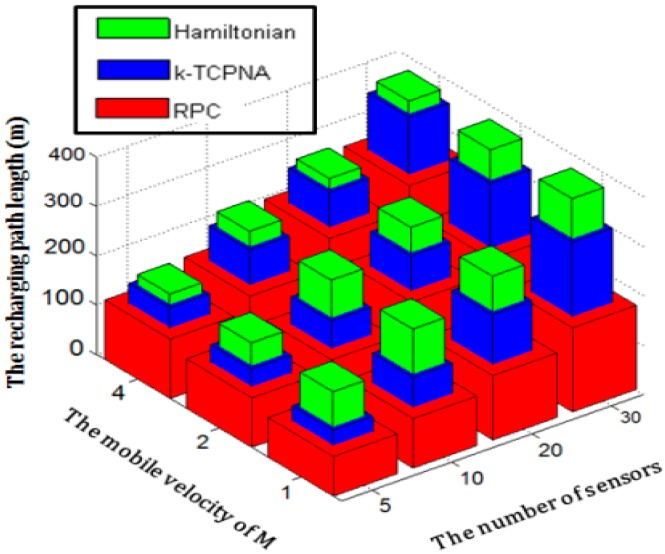
The comparison of the four mechanisms in terms of recharging path length by varying the speed of recharger from 1 to 4.

Another important parameter, the speed of mobile recharger, can impact the recharging path length. Figure 17 compares the path length of the four compared algorithms by varying the speed of mobile recharger. The number of sensors is varied from 1 to 30. As shown in Figure 17, the HAM-based recharging algorithm yields the same value of recharging path length, regardless of the speed of the mobile recharger. This occurs because that the recharging path should pass through the location of each sensor.

The change of speed of mobile recharger does not impact the paths constructed by the HAM-based and OWER-MDG mechanisms. That is, the two mechanisms construct the same paths even though the speed of mobile recharger changed. On the contrary, the length of the recharging path constructed by applying the proposed RPC is increased with the speed of the mobile recharger. This occurs because a mobile recharger with a fast speed can shorten the time period that the charger falls in the recharging range, leading to the situation that the battery of some sensors cannot be fully recharged. To guarantee that each sensor can be fully recharged, the length of the mobile recharger trip should be lengthened. In comparison, the optimal algorithm obtains the shortest recharging path. The proposed RPC outperforms the HAM-based recharging and OWER-MDG algorithms in terms of recharging path length.

## 6. Conclusions

This paper proposed an energy recharging mechanism, called RPC, which aims at achieving the perpetual operation of the WSNs while improving the efficiency of wireless energy recharge. In the proposed RPC, we consider the “recharging while moving” concept, aiming to recharge the sensors while the mobile recharger moves in their recharging range. The proposed RPC consists of four phases. In the first phase, an initial path passing through the central point of each sensor is constructed. Based on the result of the first phase, the second phase partitions the ordered sensors into different groups which are the inputs of following phases. Then the third phase establishes a recharging segment for each sensor. Moving along the segment, a mobile recharger can recharge that sensor such that sensor’s battery is guaranteed to be fully recharged. The fourth phase of the proposed RPC further reduces the path length, aiming to improve the recharging efficiency. Compared with the existing studies, the proposed RPC significantly reduces the length of the recharging path, and hence improves the recharging efficiency of WSNs while satisfying the fully recharge demands of each sensor and achieving the perpetual operation of the WSN.

## Figures and Tables

**Table 1:** The comparison between the existing algorithms and the proposed RPC.

	Charging Stability	Recharging While Moving	Without Passing Center of Sensor	Periodic Recharging
Solar system [10]	**×**	**×**	**×**	**×**
WEH system [11]	**×**	**×**	**×**	**×**
Thermal system [12]	**×**	**×**	**×**	**×**
WISP [13]	**○**	**×**	**×**	**×**
CMC [14]	**○**	**×**	**×**	**○**
DIWC system [15]	**○**	**×**	**×**	**○**
Mobile system [16]	**○**	**×**	**×**	**○**
The proposed RPC	**○**	**○**	**○**	**○**
